# In this Issue: Volume 113, Issue 4, April 2022

**DOI:** 10.1111/cas.14950

**Published:** 2022-04-08

## 1 Intranodal delivery of modified docetaxel: innovative therapeutic method to inhibit tumor cell growth in lymph nodes

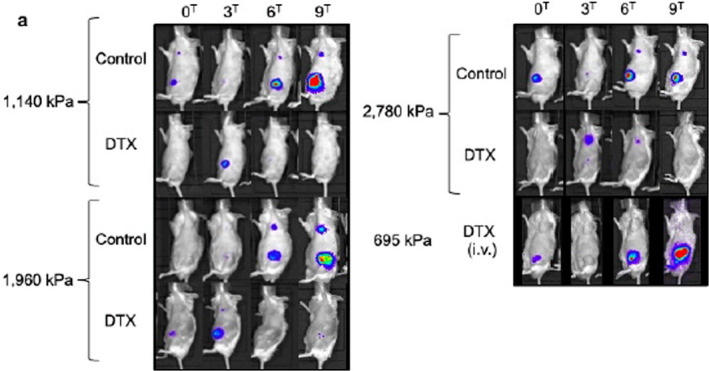

Lymph node (LN) metastasis is associated with a worse patient prognosis. Patients with positive LNs require escalation in treatment and experience increased side effects. Effective treatments of the involved LNs are required to prevent metastatic spread. Particular attention should be paid to the side effect profile as therapy can be intolerable for patients. In this study, Sukhbaatar et al utilized a LN metastatic mouse model to optimize a lymphatic drug delivery system (LDDS). They first determined the optimal osmotic pressure and viscosity ranges of the solvent for LDDS that would appropriately deliver the drug to involved LNs while minimizing systemic side effects. They then delivered a single dose of docetaxel at 10 mg/kg and were able to detect a significant antitumor effect with no treatment‐related side effects. Intranodal therapeutic delivery will eventually significantly improve outcomes and quality of life for patients with locally advanced cancer.

https://onlinelibrary.wiley.com/doi/full/10.1111/cas.15283

## 2 MEK/ERK‐mediated oncogenic signals promote secretion of extracellular vesicles by controlling lysosome function

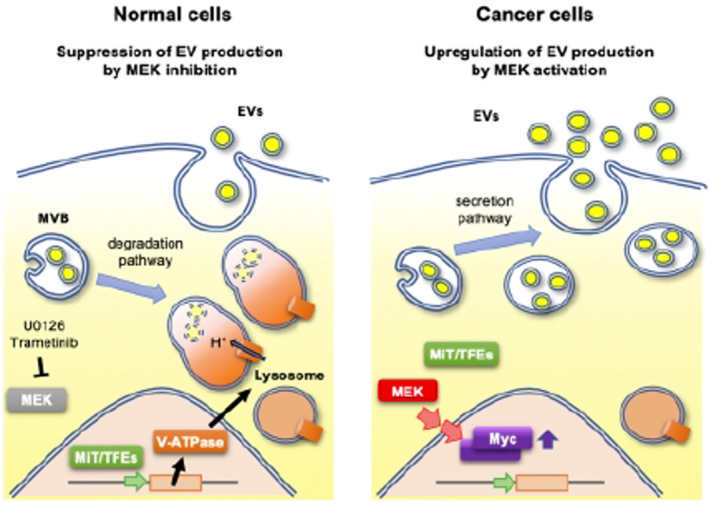

Extracellular vesicles (EVs) secretion is elevated in cancer cells. These EVs have been shown to change the local and distant tumor microenvironment, which suggests that they play a significant role in cancer metastasis. Thus, EVs have been studied for use as potential biomarkers and targets for novel therapeutics. However, the mechanism that regulates whether multivesicular bodies (MVBs) fused with lysosomes for degradation or with the plasma membrane for release as EVs is not well established. In this study, Hikita et al examined the role of mitogen‐activated protein kinase kinase (MEK) and mitogen‐activated protein kinase (ERK) on the regulation of EVs. They found that the MEK/ERK pathway inactivated lysosomal activity, which led to increased EV secretion. This increased EV secretion was regulated by MYC upregulation, which localized MiT/TFE transcription factors to the cytoplasm and suppressed transcription of lysosome‐related genes. The MEK/ERK/MYC pathway is an intriguing target to suppresses cancer cell EV secretion.

https://onlinelibrary.wiley.com/doi/full/10.1111/cas.15288

## 3 *N*‐glycosylation regulates MET processing and signaling

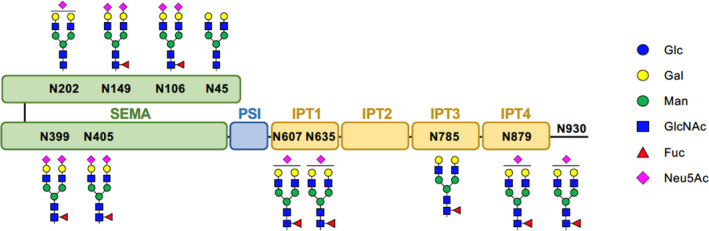

Title: N‐glycosylation regulates MET processing and signaling

Tyrosine kinase inhibitors (TKIs) have been extremely effective treatments for lung cancer patients with targetable mutations. The one caveat is that these patients will eventually develop resistance to the TKIs. MET, a receptor tyrosine kinase (RTK) for hepatocyte growth factor (HGF), has been studied because amplification of MET has been associated with TKI resistance. However, there are very few drugs that have any clinical indications. In this study, Saitou et al examined the role that *N*‐glycosylation played in the regulation of MET. They found that inhibition of *N*‐glycosylation suppressed the processing and trafficking of endogenous MET. Furthermore, the deletion of *N*‐glycan of MET showed similar results and suggested that the SEMA domain played an important role in *N*‐glycan mediated regulation of MET. Targeting *N*‐glycosylation may produce an efficacious MET targeted therapeutic.

https://onlinelibrary.wiley.com/doi/full/10.1111/cas.15278

